# Supplementary material for: SyNDI: synchronous network data integration framework
Source: BMC Bioinformatics. 2018 Nov 6;19:403. doi: 10.1186/s12859-018-2426-5 (PMC6219086; doi:10.1186/s12859-018-2426-5)
Supplement: Supplementary file 5 — Files of the example presented in Additional file 4. This additional file is a zip package that contains all files used in Additional file 4. Detailed descriptions of each of these files are presented in a README file included in this zip package. (GZ 30411 kb) [file 12859_2018_2426_MOESM5_ESM.gz › SyNDITools/Meme2Fimo/meme_4.9.0_4.tar.gz!/meme_4.9.0/website/html/submit-verify.in]

Meta-MEME v3.3 - Data submission


  
  
  
  
  

### Data submission Form

Use this form to submit DNA or protein sequences to Meta-MEME. In
addition to your sequences, Meta-MEME requires as input a MEME motif analysis of your data in HTML format. Using
the output from MEME, Meta-MEME will build a motif-based hidden
Markov model of your sequences and will use the model to
 search
a sequence database for homologous sequences.

You will be directed to a URL that contains the links to
your results.

---

### Inputs

Please enter the following inputs:  


1. The name of a file containing a set of MEME motifs from your sequences. This
   file will be used to build the initial hidden Markov model.
2. **[Optional]**The name of a file containing a set of
    DNA or  protein sequences
   in an acceptable format.
3. **[Optional]** Description of your sequences:
4. **[Optional]** The sequence database you want to search:  

   Protein Databank


   Yeast (S. cerevisiae)
   genome
   C. elegans
   genome
   E. coli genome
   SWISS-PROT (protein
   only)
   Genpept (protein
   only)

   NCBI non-redundant STS database (DNA
   only)
   Vector subset of Genbank (DNA
   only)
   Eukaryotic Promoter Database (DNA
   only)

  

   


---

### Outputs

Please select the desired output(s).  

1. A motif-based hidden Markov model.
2. An analysis of the motif occurrences in your
   sequences.
3. A list of homologous sequences from the
   selected database.

  


---

    


---

Return to the Meta-MEME home page.  
  
*Please send comments and questions to @METAMEME\_CONTACT@*
